# MR-Based Radiomics for Differential Diagnosis between Cystic Pituitary Adenoma and Rathke Cleft Cyst

**DOI:** 10.1155/2021/6438861

**Published:** 2021-08-10

**Authors:** Yanping Wang, Sixuan Chen, Feng Shi, Xiaoqing Cheng, Qiang Xu, Jianrui Li, Song Luo, Pengbo Jiang, Ying Wei, Changsheng Zhou, Lijuan Zheng, Kaiwei Xia, Guangming Lu, Zhiqiang Zhang

**Affiliations:** ^1^Department of Diagnostic Radiology, Jinling Hospital, Medical School of Nanjing University, Nanjing 210002, China; ^2^Department of Research and Development, Shanghai United Imaging Intelligence Co., Ltd., Shanghai 200232, China; ^3^State Key Laboratory of Analytical Chemistry for Life Science, Nanjing University, Nanjing 210093, China

## Abstract

**Background:**

It is often tricky to differentiate cystic pituitary adenoma from Rathke cleft cyst with visual inspection because of similar MRI presentations between them. We aimed to design an MR-based radiomics model for improving differential diagnosis between them.

**Methods:**

Conventional diagnostic MRI data (T1-,T2-, and postcontrast T1-weighted MR images) were obtained from 215 pathologically confirmed patients (105 cases with cystic pituitary adenoma and the other 110 cases with Rathke cleft cyst) and were divided into training (*n* = 172) and test sets (*n* = 43). MRI radiomics features were extracted from the imaging data, and semantic imaging features (*n* = 15) were visually estimated by two radiologists. Four classifiers were used to construct radiomics models through 5-fold crossvalidation after feature selection with least absolute shrinkage and selection operator. An integrated model by combining radiomics and semantic features was further constructed. The diagnostic performance was validated in the test set. Receiver operating characteristic curve was used to evaluate and compare the performance of the models at the background of diagnostic performance by radiologist.

**Results:**

In test set, the combined radiomics and semantic model using ANN classifier obtained the best classification performance with an AUC of 0.848 (95% CI: 0.750-0.946), accuracy of 76.7% (95% CI: 64.1-89.4%), sensitivity of 73.9% (95% CI: 56.0-91.9%), and specificity of 80.0% (95% CI: 62.5-97.5%) and performed better than multiparametric model (AUC = 0.792, 95% CI: 0.674-0.910) or semantic model (AUC = 0.823, 95% CI: 0.705-0.941). The two radiologists had an accuracy of 69.8% and 74.4%, respectively, sensitivity of 69.6% and 73.9%, and specificity of 70.0% and 75.0%.

**Conclusions:**

The MR-based radiomics model had technical feasibility and good diagnostic performance in the differential diagnosis between cystic pituitary adenoma and Rathke cleft cyst.

## 1. Introduction

Cystic pituitary adenoma (CPA) and Rathke cleft cyst (RCC) are both common intrasellar cystic lesions, but they have different treatment strategies and prognosis in clinic [1–4]. Although endocrinous test is effective for differentiating these two diseases, MRI is still the vital tool in diagnosis of these two lesions, especially in the case of nonfunctional pituitary adenoma. MR image features for CPA and RCC have been well documented over these years, for example, features of fluid-fluid level, off-midline location, septation, and hypointense rim on T2-weighted images (T2WI) are rather specific for CPA, and intracystic nodule is commonly seen in RCC [5–10]. In many cases, these two lesions were difficultly distinguished on MRI manifestations. CPA can present high- (subacute bleeding) or low- (liquefaction) intensity on T1-weighted imaging (T1WI) and can also present high (liquefaction) or low (necrosis underpinned by chronic bleeding) intensity on T2WI. These radiological manifestations can imitate those of the RCC with various intracystic protein levels [5, 11–13]. Thus, the overlapped imaging manifestations pose challenge for differential diagnosis with visual inspection.

Recently, computer-aided diagnosis and quantitative imaging analysis have been increasingly applied to MRI of intrasellar lesions [14–16]. As a natural extension of computer-aided diagnosis, radiomics has become a promising technique for diagnosis in radiological field [17] and has been applied to differential diagnosis in many diseases of whole body [18–21]. In contrast to conventional computer-aided diagnosis, radiomics has advantages in high-throughput features and mineable data that may improve diagnostic accuracy [17, 22]. Previous research has established that the combination of signal intensity on the postcontrast image and texture features can be used to discriminate pituitary adenoma and RCC, based on the fact that pituitary adenoma was more likely to show enhancement while RCC rarely showed enhancement on the contrast-enhanced image [15]. In our opinion, pituitary adenomas with solid enhancement can be well distinguished from RCC, but it is difficult to differentiate CPA with nonenhancement or thin-rim enhancement from RCC. In this study, we aimed to estimate the diagnostic capability of MR-based radiomics model in differentiating CPA and RCC.

## 2. Materials and Methods

### 2.1. Patient Selection

This retrospective study was approved by our institutional review board, and informed consent was waived. A total of 230 patients with an imaging presentation of cystic lesion on preoperative contrast-enhanced MR examination and pathological confirmation of pituitary adenoma (*n* = 117) and RCC (*n* = 113) were consecutively collected from July 2009 to February 2021. Patients were excluded due to low-quality or incomplete MRI data (*n* = 12), a history of surgery, and radiotherapy in the sellar region (*n* = 3). This study finally included 215 patients, including 105 patients with CPA and 110 patients with RCC. All of 215 patients were divided into two nonoverlapping set according to the MR images acquisition time: 172 cases (82 CPAs and 90 RCCs) who underwent MRI scan during period from July 2009 to June 2019 were assigned as training set, and the remaining 43 cases (23 CPAs and 20 RCCs) who were scanned from July 2019 to February 2021 were set as test set.

### 2.2. Image Acquisition

MRI data were obtained on four scanners in our hospital (3.0 T Siemens Trio, 3.0 T GE Discovery MR750, 1.5 T GE Signa, and 1.5 T Siemens Magnetom) with the following protocols: (1) sagittal T1WI (TR, 360-450 msec; TE, 13-17 msec; section thickness, 2-3 mm; matrix, 256 × 192; and FOV, 20 × 20 cm), coronal T1WI (TR, 400-440 msec; TE, 10-17 msec; section thickness, 2-3 mm; matrix, 256 × 179; and FOV, 20 × 20 cm), coronal T2WI (TR, 3500-4000 msec; TE, 92-113 msec; section thickness, 2-3 mm; matrix, 320 × 240; and FOV, 20 × 20 cm). Sagittal and coronal postcontrast T1WI were performed after an intravenous bolus injection (0.1 mL/kg) of gadolinium-based contrast (gadopentetate dimeglumine).

### 2.3. Region-of-Interest (ROI) Segmentation

A radiologist (with 5 years of experience) manually delineated the ROI along the boundary of the entire lesion on coronal T2WI and coronal postcontrast T1WI layer by layer using an open-source MRICRON software (version 6). Another senior radiologist (with 10 years of experience) examined the outline results. To transfer the segmentations to the T1 sequence, the postcontrast T1 sequence were linearly aligned to the T1 sequence using SPM12 software package on the platform of MATLAB (http://www.fil.ion.ucl.ac.uk/spm/software/spm12), thereby compensating for patient movement between the two scans.

### 2.4. Radiomics Feature Extraction

Both T1WI, T2WI, and postcontrast T1WI data were subjected to radiomics feature extraction by using Pyradiomics software [23]. Feature normalization was performed before the estimation of radiomics features. The 110 radiomics features were extracted from each of T1WI, T2WI, and postcontrast T1WI data, and comprised seven groups: 19 first-order statistical features, 17 shape-based features, 23 gray level cooccurrence matrix (GLCM) features, 16 gray level run length matrix (GLRLM) features, 16 gray level size zone matrix (GLSZM) features, 5 neighbouring gray tone difference matrix (NGTDM) features, and 14 gray level dependence matrix (GLDM) features. Thus, a total of 330 radiomics features were extracted from the original images. The first-order statistical features can evaluate the attributes of the individual pixel value, but are independent of spatial interaction between pixels [24]. The shape-based features are morphological properties such as volume and size. The remaining features belong to texture features and can be used to characterize irregularity of tissues [25].

### 2.5. Semantic Feature Evaluation

**Figure 1:**
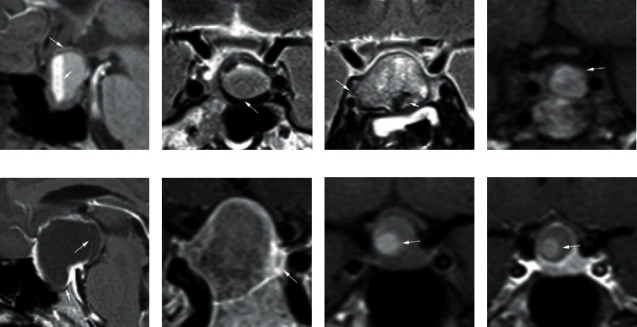
Semantic features for image analysis ((a–f): patients with CPA; (g–h): patients with RCC): (a) fluid-fluid level (short arrow) and wall thickness nonuniformity (long arrow) (sagittal T1 image); (b) a hypointense rim on T2WI (coronal T2 image); (c) heterogeneous of cystic portion (short arrow) and beyond the lateral margin of the cavernous ICA (long arrow) (coronal T2 image); (d) off-midline location (coronal T1 image); (e) sellar floor depression (short arrow) and intracapsular septation (long arrow) (sagittal postcontrast T1 image); (f) ill-defined lesion boundary (coronal postcontrast T1 image); (g) intracystic nodule (coronal T1 image); (h) intracystic nodule (coronal postcontrast T1 image).

Two radiologists (radiologist 1 with 10 years of working experience, radiologist 2 with 15 years of working experience) who were blinded to clinical information and pathologic results independently reviewed the MR images to evaluate semantic features for all patients. The semantic features included: (1) tumor shape (round, oval, snowman-like, and lobulated [7]); (2) tumor location (intrasellar, intrasellar and suprasellar, suprasellar); (3) sellar floor depression (defined as a sellar floor depth exceeding 10 mm below the imaginary posterior extension line from the planum sphenoidale [26]; absence/presence); (4) intensity on T1WI (defined the white matter of the brain as the reference standard; divided into 6 groups: hypointensity, iso-hypointensity, isointensity, iso-hyperintensity, hyperintensity, or hyperhypointensity); (5) intensity on T2WI (classified as above); (6) off-midline location (defined as lateralization of the lesion in the sella turcica or stalk deviation by the lesion [6, 7], absence/presence); (7) signal intensity of cystic portion (homogeneous/heterogeneous); (8) cyst wall thickness (uniformity/nonuniformity); (9) lesion boundary (well-defined/ill-defined); (10) inner margin of cyst wall (regular, irregular); (11) fluid-fluid level [9] (absence/presence); (12) intracapsular septation [6] (absence/presence); (13) a hypointense rim on T2WI (the peripheral portion of a sellar lesion was lower than the intensity of white matter on T2WI [5, 6], absence/presence); (14) intracystic nodule (free-floating nodules without enhancement [8], absence/presence); and (15) the relationship with the cavernous sinus (defined by whether within or beyond the lateral margin of the cavernous intracranial carotid artery (ICA) [7], within/beyond) (Figure 1). In case of disagreement in semantic feature evaluation, consensus was achieved by disscusion. If consensus still could not be achieved, a senior neuroradiologist (with more than 18 years of experience) assisted to reach a consensus. The results were summarized in Supplementary Table (available here). Meanwhile, the two radiologists were informed that the final diagnosis was one of the two tumors (CPA or RCC), and they separately diagnosed all the cases.

### 2.6. Feature Selection and Classifier Training

**Figure 2:**
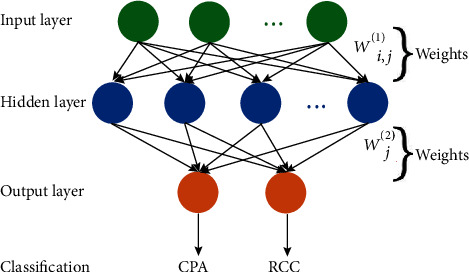
Illustration of ANN architecture. The input layer includes a number of input nodes. Then, *w*_*ij*_^(1)^ denotes the weights that connect the *i*th input to the *j*th node in the hidden layer. *w*_*j*_^(2)^ is the weight that connects the *j*th hidden neuron to the output layer neuron.

The feature selection and classification method were computed using sklearn (https://scikit-learn.org/stable/).To avoid collinearity and overfitting in feature space, least absolute shrinkage and selection operator (LASSO) algorithm was used for feature selection [27, 28]. To assess the predictive value of the radiomics features and semantic features, three models were trained and tested based on radiomics features only (multiparametric model), semantic features only (semantic model), and a combination of radiomics features and semantic features (the combined radiomics and semantic model). Furthermore, out of curiosity about the differences in the discriminative ability of each single parametric imaging feature in multiparameter model, we compared the diagnostic value of models based on single parametric imaging feature: T1 imaging features only (T1WI model), T2 imaging features only (T2WI model), and postcontrast T1 imaging features only (postcontrast T1WI model). For classification, we investigated four machine learning classifiers, including support vector machine (SVM), artificial neural network (ANN), adaptive boosting (AdaBoost), and random forest (RF). SVM learns an optimal hyperplane that separates the classes as wide as possible, while trying to balance with misclassified cases [29]. For SVM model, a radial basis function (RBF) ke, rnel is used, together with regularization parameter *C* of 1.0. ANN, inspired by biological neural networks, has a remarkable self-learning ability to investigate the meaning and rules of complicated data [30, 31]. For ANN model, a three-layer feedback architecture (i.e., one input layer, one hidden layer with 100 neurons, and one output layer) was performed (Figure 2). ReLU transfer function was used in the hidden and output layers. Adam optimization algorithm was adopted to update the network weights. The overfit penalty and maximum iteration number were set as 0.0001 and 200, respectively. We also used RF and AdaBoost, two tree-based ensemble learning classifiers that allow nonlinear interactions between features and have good interpretability, to develop our models. For AdaBoost and RF, the classification models were trained with the number of trees as 100, maximum depth as 10.

**Figure 3:**
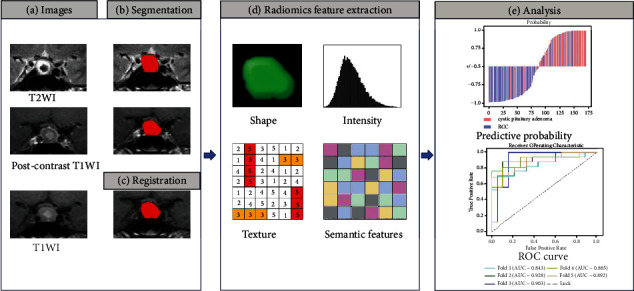
Workflow of radiomics approach. (a) Input T1, T2, and postcontrast T1 images. (b) Segmentation of the tumor on the T2 image and postcontrast T1 image. (c) Registration of the postcontrast T1 to the T1 image to transform this segmentation to the T1 image. (d) Feature extraction from the T1, T2, and postcontrast T1 images, combined with semantic features. (e) Predictive analysis and model evaluation.

To compare the performance of models, we computed different combinations of feature selection methods and classifiers. A schematic overview of the radiomics approach is shown in Figure 3.

### 2.7. Statistical Analyses

#### 2.7.1. Group Comparison

All statistical analyses were performed using SPSS software, v.21(IBM Corp, Armonk, New York, USA). The demographic and clinical characteristics were compared by a *χ*^2^ test for categorical variables and a Kolmogorov-Smirnov test for continuous variables. A two-sample *t*-test or a nonparametric *t*-test was applied to analyze the statistical differences, depending on whether they conform to the normal distribution of continuous variables. *P* values less than 0.05 was considered statistically significant.

#### 2.7.2. Model Construction and Evaluation

All classification experiments based on different combinations of feature selection and machine learning classifiers were performed through 5-fold crossvalidation, and the whole training set was randomly divided into five subsets. In each fold, four subsets were used as the training set, and the rest one subset was used as the testing set. In the training stage, LASSO was used to select the most relevant features. Due to the different training set, the number of selected features ranged between 20 and 40. After that, cluster models were trained based on these features in the training set. In the testing stage, these trained models were applied to the testing set, and the classification results were obtained. This process was repeated until all subsets served as the testing set once. Four machine learning classifiers were utilized, and parameters with the best AUC were selected for each model. Then, the radiomics model, semantic model, and the combined radiomics and semantic model were externally validated in the test set.

#### 2.7.3. Comparison of Diagnostic Performance

The diagnostic performance of the six models was calculated using AUC in the training set. The multiparametric model, semantic model, and the combined radiomics and semantic model was evaluated and compared using AUC, accuracy, sensitivity, specificity, positive predictive value, and negative predictive value in the training and test set. Additionally, we assessed the diagnostic performance of the radiologists who had previously evaluated semantic features and distinguished CPA from RCC.

## 3. Results

### 3.1. Patient Characteristics

Clinical and demographic characteristics were summarized in Table 1. In either the training or the test set, there were no significant statistical differences in age and gender between the two groups. Abnormal hormone level occurred more frequently in CPAs than in RCCs in both the training (*P* < 0.001) and the test (*P* = 0.01) sets (Table 1).

### 3.2. Determination of the Best Models in the Training Set

The results of the diagnostic performance using different combinations of six feature-selection methods and four classifiers in the training set were summarized in Table 2.

#### 3.2.1. The Models Based on Radiomics Features

The ANN classifier achieved consistent superior performance with at least 2.3% higher AUC over other classifiers except using T1 imaging features. The ANN classifier showed the best diagnostic performance in multiparametric model (mean AUC = 0.890, 95% CI: 0.851-0.929) than that of other radiomics models. The ANN classifier showed the best diagnostic performance in T2WI model (mean AUC = 0.847) and postcontrast T1WI model (mean AUC = 0.867) than that of other classification methods. The performance of the SVM or RF classifier (mean AUC = 0.756) was better than that of the other two classification methods in T1WI model.

#### 3.2.2. The Semantic Model

The ANN classifier showed the best diagnostic performance (mean AUC = 0.902, 95% CI: 0.863-0.941) in semantic model than that of other classification methods.

#### 3.2.3. The Combined Radiomics and Semantic Model

The ANN classifier in the combined radiomics and semantic model achieved the best diagnostic performance (mean AUC = 0.924, 95% CI: 0.885-0.963) than that of other classification methods.

### 3.3. Comparison of the Models in the Training and Test Set

The combined radiomics and semantic model had better diagnostic performance than either the multiparametric model or the semantic model in the training and test set (Table 3).

**Figure 4:**
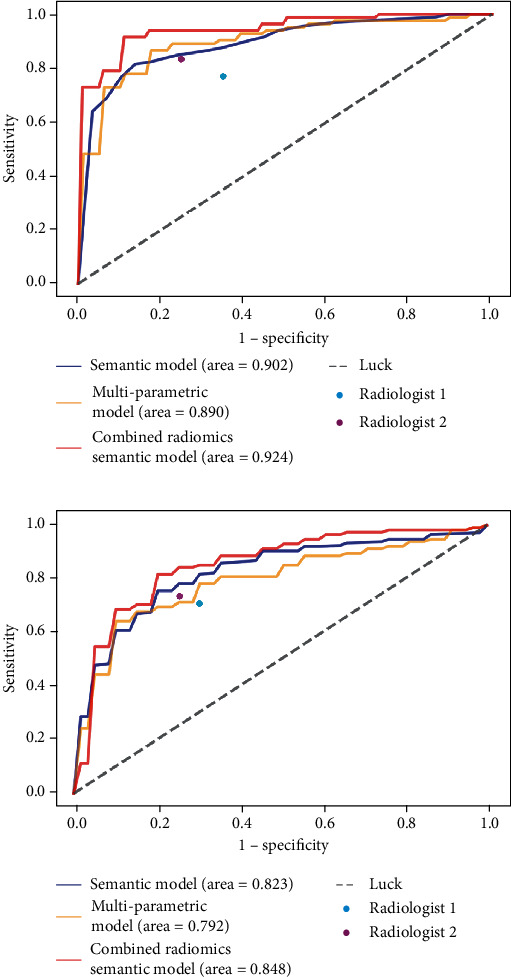
ROC curves for ANN classifier with multiparametric model, semantic model, and combined radiomics and semantic model in training set (a) and test set (b). The performances of the radiologists are also shown with red and blue dots.

The ANN classifier in combined radiomics and semantic model yielded an AUC, accuracy, sensitivity, and specificity of 0.924, 85.5%, 86.6%, and 84.4% for the training set and 0.848, 76.7%, 73.9%, and 80.0% for the test set (Table 3). Additionally, the ROC curves were shown in Figure 4.

### 3.4. Diagnostic Performance of Radiologists

The radiologists achieved an accuracy of 70.9% and 79.1%, respectively, sensitivity of 76.8% and 82.9%, and specificity of 65.6% and 75.6% in the training set. In the test set, two radiologists had an accuracy of 69.8% and 74.4%, respectively, sensitivity of 69.6% and 73.9%, and specificity of 70.0% and 75.0% (Figure 4).

### 3.5. Model Analysis

**Figure 5:**
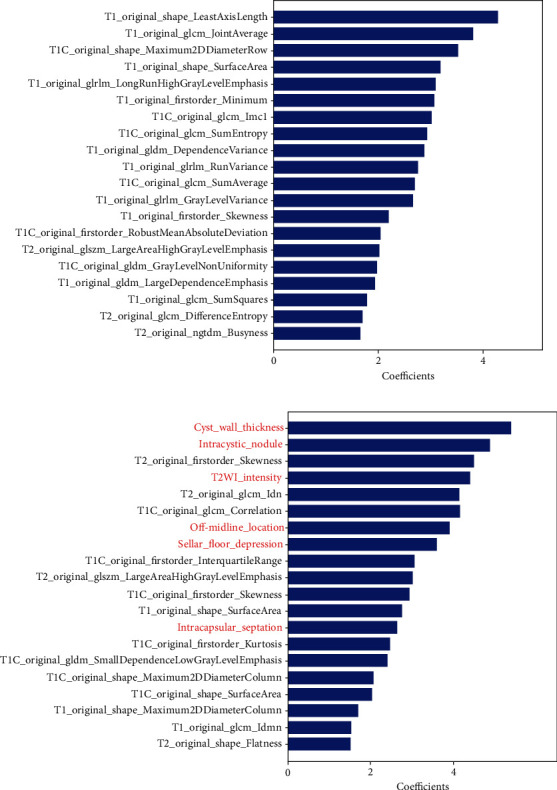
TOP 20 importance ranking of features in multiparametric model (a) and combined radiomics and sematic model (b) by LASSO in 5 folds in training set. Features with name starting with “T1C_original” are radiomics features extracted from postcontrast T1WI; “T2_original” are radiomics features extracted from T2WI; “original” are radiomics features extracted from T1WI, and the others are sematic features (marked in red).

In the training set, we computed the importance ranking of features that is selected by LASSO regression with the optimal lambda including nonzero variables in each round of crossvalidation and mixed them up to select the TOP20 importance ranking of features (Figure 5). For multiparametric model, the selected features were mainly derived from texture features, such as entropy, emphasis, and nonuniformity. The TOP 20 features in multiparametric model included 3 first-order features, 3 shape-based features, 6 GLCM features,3 GLRLM features, 1 GLSZM features, 1 NGTDM features, and 3 GLDM features (Figure 5).

In the training set, the TOP 20 importance feature selection in the combined radiomics and semantic model, 6 semantic features and 14 radiomics features, was found retained after LASSO feature selection in 5-fold (Figure 5). The 6 semantic features were sellar floor depression, T2WI intensity, off-midline location, cyst wall thickness, intracapsular septation, and intracystic nodules. The selected radiomics features mainly comprised first-order features and shape-based features. The14 radiomics features included 4 first-order features, 5 shape-based features, 3 GLCM features, 1 GLSZM features, and 1 GLDM features. Among the TOP 20 features of the two models, the overlapping feature is original_shape_SurfaceArea.

**Figure 6:**
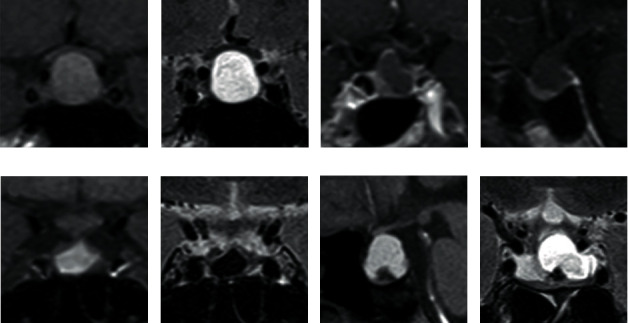
Examples of cases who were classified incorrectly by the combined radiomics and semantic model: (a, b) patients with CPA (coronal T1 and T2 image); (c, d) patients with CPA (coronal and sagital postcontrast T1 image); (e, f) patients with RCC (coronal T1 and T2 image); (g, h) patients with RCC (sagital T1 image and coronal T2 image).

Among the 43 cases in the test set, 10 cases (4 RCC, 6 CPA) were classified incorrectly by the combined radiomics and semantic model. Some of the misclassified cases were shown in Figure 6.

## 4. Discussion

In this study, we used a radiomics-based machine learning method to distinguish CPA from RCC. Our results indicated that the radiomics analysis based on traditional MR images provide a promising noninvasive method and yield better diagnostic performance than radiologists. Another important finding was that the performance of combined radiomics and semantic model has been further improved on the basis of adding semantic features.

The diagnostic accuracy of the radiomics model was higher than the experienced radiologists. As we know, the diagnostic performance of radiologists is based on their experience and subjective perception of conventional MR image, while radiomics approach could discover subtle differences that were not perceptible by visual inspection and allow for reproducible analysis [17, 22, 32].

This study was designed to compare the diagnostic efficacy of radiomics models based on different single parametric image. Consistent with the prior study of Zhang et al. [15], this research found that the most significant MR image data in single parametric model for differential diagnosis is postcontrast T1 image, followed by T2 image. However, T1 hyperintensity can be seen in intratumoral hemorrhage in PA and high concentrations of protein in RCC, which may be mistaken for contrast-enhanced tumors; the evaluation of postcontrast T1WI alone may be misleading [13, 33]. Thus, we calculated the performance of multiparametric model and found that multiparametric images had more contribution to improve the performance. In multiparametric model, the selected importance features were mainly derived from texture features; entropy and uniformity could be used to quantified heterogeneity at relevant scales [34]. Entropy reflected the texture irregularity, while uniformity represented the distribution of gray levels within the tumor [34]. The heterogeneity may be correlated with specific radiographic signs, such as the fluid-fluid level, the septation, and floating nodule, which affect the texture characteristics.

Considering that semantic features are important in differential diagnosis and may provide additional predictive value, we added it to radiomics features to build the integrated model. As we expected, the combined radiomics and semantic model performed better than the model based on radiomics or semantic features alone. In addition to valuable semantic features consistent with previous studies, we tried to explain the importance of sellar floor depression and cyst wall thickness in the selected importance features. We speculated that sellar floor depression may be related to the invasiveness of PA, and PA could infiltrate many structures such as the sellar floor, the cavernous sinus, and the suprasellar region [35–37]. However, the growth of RCC was expansive and noninvasive behavior pattern. The main MRI finding of RCC was nonenhancement or thin-rim enhancement. Earlier studies have shown that the thin-rim enhancement of RCC can be attributed to squamous metaplasia, inflammation, deposition of hemosiderin, or cholesterol crystals in the cyst wall [38–41]. Therefore, it is important to distinguish that RCC is surrounded by enhanced normal pituitary gland to simulate the enhancement of cyst wall enhancement [41, 42]. The wall of CPA is attributed to the incomplete hemorrhage, infarction, or hemorrhagic infarction occurred in the solid part of pituitary adenoma, so the thickness of the wall can be nonuniformity. The radiomics features in the selected importance features mainly composed of first-order features and shape-based features. The correlation analysis between semantic features and texture remains to be studied.

In the test set, we found that the performance of the three models decreased compared to training set, but still had relatively good performance compared to radiologists. Different manufacturers and different parameter settings may be a factor affecting the image quality and manifestation; thus, we speculated that this may be an impact on the performance of the models, whereas, from another point of view, it was proved that the radiomics techniques had rather superior generalization performance even thought it was constructed with heterogeneous data.

There were several limitations in our study. First, the relatively small number of patients in test set in this study might influence our results, and multicenter data might be needed to validate our model in the future. Second, this study did not include highly suspected patients without surgery, which may lead to bias in the results.

## 5. Conclusions

The radiomics approach was a feasible method to distinguish CPA from RCC, and the diagnostic performance of radiomics model outperformed radiologists. The performance of the model was further improved after semantic features were added. The combined radiomics and semantic model utilizing the ANN classifier was considered to be the optimal model for identifying CPA and RCC.

## Figures and Tables

**Table 1:** Clinical and demographic characteristics of patients.

	Training set (*n* = 172)		*P* value	Test set (*n* = 43)		*P* value
	CPA (*n* = 82)	RCC (*n* = 90)		CPA (*n* = 23)	RCC (*n* = 20)	
Age (mean ± SD), y	43.39 ± 14.27	42.23 ± 14.35	.597^b^	40.43 ± 15.97	47.65 ± 16.18	.150^b^
Gender, male/female ratio	31 : 51	37 : 53	.658^a^	7 : 16	8 : 12	.512^a^
Abnormal hormone level, *n* (%)	77 (93.9)	55 (61.1)	<.001^a^	17 (73.9)	10 (35.0)	.010^a^
Hormonal symptoms *n* (%)						
With	24 (29.3)	7 (7.8)		8 (34.8)	3 (15.0)	
Without	58 (70.7)	83 (92.2)	<.001^a^	15 (65.2)	17 (85.0)	.138^a^
Visual loss *n* (%)						
With	37 (45.1)	23 (25.6)		7 (30.4)	3 (15.0)	
Without	45 (54.9)	67 (74.4)	.007^a^	16 (69.6)	17 (85.0)	.232^a^

Note: SD indicates standard deviation. Data in parentheses are percentages. a: from the *χ*^2^ test; b: from the two independent sample *t*-tests.

**Table 2:** The mean AUC value of fivefold crossvalidation using different combinations of feature selection and classifiers in the training set.

Model	Classifier			
	ANN	SVM	AdaBoost	RF
T1WI model	0.722	0.756	0.682	0.756
T2WI model	0.847	0.835	0.779	0.817
Postcontrast T1WI model	0.867	0.850	0.829	0.847
Multiparametric model	0.890	0.889	0.845	0.868
Semantic model	0.902	0.842	0.844	0.873
Combined radiomics and semantic model	0.924	0.907	0.849	0.889

**Table 3:** Comparison of diagnostic performance of the semantic model, multiparametric model, and combined radiomics and semantic model using ANN classifier in the training and test set.

*Training set*	*AUC*	*Sensitivity*	*Specificity*	*Accuracy*	*PPV*	*NPV*
Semantic model	0.902 [86.3, 94.1]	0.756 [66.3, 84.9]	0.933 [88.2, 98.5]	0.849 [79.5, 90.2]	0.912 [84.4, 97.9]	0.808 [73.2, 88.3]
Multiparametric model	0.89 [85.1, 92.9]	0.793 [70.5, 88.0]	0.844 [77.0, 91.9]	0.820 [76.2, 87.7]	0.823 [73.9, 90.7]	0.817 [73.9, 89.6]
Combined radiomics and semantic model	0.924 [88.5, 96.3]	0.866 [79.2, 94.0]	0.844 [77.0, 91.9]	0.855 [80.2, 90.7]	0.835 [75.6, 91.4]	0.874 [80.4, 94.3]

*Test set*	*AUC*	*Sensitivity*	*Specificity*	*Accuracy*	*PPV*	*NPV*
Semantic model	0.823 [70.5, 94.1]	0.783 [61.4, 95.1]	0.850 [69.4, 100.0]	0.814 [69.8, 93.0]	0.857 [70.7, 100.0]	0.773 [59.8, 94.8]
Multiparametric model	0.792 [67.4, 91.0]	0.522 [31.8, 72.6]	0.900 [76.9, 100.0]	0.698 [56.0, 83.5]	0.857 [67.4, 100.0]	0.621 [44.4, 79.7]
Combined radiomics and semantic model	0.848 [75.0, 94.6]	0.739 [56.0, 91.9]	0.800 [62.5, 97.5]	0.767 [64.1, 89.4]	0.810 [64.2, 97.7]	0.727 [54.1, 91.3]

Note: data in parentheses are 95% confidence intervals. PPV: positive predict value; NPV: negative predict value.

## Data Availability

The datasets used and/or analyzed during the current study are available from the corresponding author on reasonable request.

## Supplementary Materials

Supplementary Materials Supplementary material: a statistical table for the semantic features of the training and test set. Note: data in parentheses are percentages. ^∗^From the *χ*^2^ test; ^∗∗^from the Fisher's exact test; ^∗∗∗^from the *χ*^2^ test with continuity correction.
