# Supplementary material for: MR-Based Radiomics for Differential Diagnosis between Cystic Pituitary Adenoma and Rathke Cleft Cyst
Source: Comput Math Methods Med. 2021 Aug 10;2021:6438861. doi: 10.1155/2021/6438861 (PMC8373489; doi:10.1155/2021/6438861)
Supplement: Supplementary Materials — Supplementary material: a statistical table for the semantic features of the training and test set. Note: data in parentheses are percentages. ∗From the χ2 test; ∗∗from the Fisher's exact test; ∗∗∗from the χ2 test with continuity correction. [file 6438861.f1.docx]

Supplementary Table S1: A statistical table for the semantic features of the training and test set.

| MRI semantic features |  | Training set | | P value | Test set | | P value |
| --- | --- | --- | --- | --- | --- | --- | --- |
|  |  | CPA(n=82) RCC(n=90) | |  | CPA(n=23) RCC(n=20) | |  |
| tumor shape | round | 8(9.8) | 15(16.7) | .030* | 0(0.0) | 4(20.0) | .028** |
|  | oval | 30(36.6) | 40(44.4) |  | 11(47.8) | 12(60.0) |  |
|  | snowman-like | 25(30.5) | 28(31.1) |  | 9(39.1) | 4(20.0) |  |
|  | lobulated | 19(23.2) | 7(7.8) |  | 3(13.0) | 0(0.0) |  |
| tumor location | intrasellar | 19(23.2) | 35(38.9) | <.001** | 8(34.8) | 13(65.0) | .044** |
|  | intrasellar and suprasellar | 63(76.8) | 47(52.2) |  | 15(65.2) | 6(30.0) |  |
|  | suprasellar | 0(0) | 8(8.9) |  | 0(0.0) | 1(5.0) |  |
| sellar floor depression | without | 27(32.9) | 68(75.6) | <.001* | 14(60.9) | 20(100.0) | .006*** |
|  | with | 55(67.1) | 22(24.4) |  | 9(39.1) | 0(0.0) |  |
| intensity on T1WI | hypointensity | 18(22) | 13(14.4) | .001* | 2(8.7) | 3(15.0) | .572** |
|  | isointensity | 8(9.8) | 24(26.7) |  | 4(17.4) | 1(5.0) |  |
|  | hyperintensity | 4(4.9) | 16(17.8) |  | 5(21.7) | 6(30.0) |  |
|  | iso-hypointensity | 19(23.2) | 9(10) |  | 1(4.3) | 1(5.0) |  |
|  | iso-hyperintensity | 26(31.7) | 24(26.7) |  | 10(43.5) | 6(30.0) |  |
|  | hyper-hypointensity | 7(8.5) | 4(4.4) |  | 1(4.3) | 3(15.0) |  |
| intensity on T2WI | hypointensity | 1(1.2) | 2(2.2) | <.001** | 1(4.3) | 3(15.0) | .555** |
|  | isointensity | 1(1.2) | 7(7.8) |  | 3(13.0) | 3(15.0) |  |
|  | hyperintensity | 10(12.2) | 23(25.6) |  | 3(13.0) | 3(15.0) |  |
|  | iso-hypointensity | 1(1.2) | 15(16.7) |  | 2(8.7) | 3(15.0) |  |
|  | iso-hyperintensity | 33(40.2) | 16(17.8) |  | 9(39.1) | 3(15.0) |  |
|  | hyper-hypointensity | 36(43.9) | 27(30) |  | 5(21.7) | 5(25.0) |  |
| off-midline location | off-midline location | 18(22) | 15(16.7) | .379* | 13(56.5) | 3(15.0) | .005* |
|  | midline location | 64(78) | 75(83.3) |  | 10(43.5) | 17(85.0) |  |
| signal intensity of cystic portion | heterogeneous | 58(70.7) | 58(64.4) | .379* | 15(65.2) | 13(65.0) | .988* |
|  | homogeneous | 24(29.3) | 32(35.6) |  | 8(34.8) | 7(35.0) |  |
| cyst wall thickness | nonuniformity | 60(73.2) | 3(3.3) | <.001* | 13(56.5) | 1(5.0) | <.001* |
|  | uniformity | 22(26.8) | 87(96.7) |  | 10(43.5) | 19(95.0) |  |
| lesion boundary | ill-defined | 30(36.6) | 0(0) | <.001* | 7(30.4) | 0(0.0) | <.001* |
|  | well-defined | 52(63.4) | 90(100) |  | 16(69.6) | 20(100.0) |  |
| inner margin of cyst wall | without | 54(65.9) | 88(97.8) | <.001* | 19(82.6) | 20(100.0) | .152*** |
|  | with | 28(34.1) | 2(2.2) |  | 4(17.4) | 0(0.0) |  |
| fluid-fluid level | without | 72(87.8) | 90(100) | .002*** | 18(78.3) | 20(100.0) | .082*** |
|  | with | 10(12.2) | 0(0) |  | 5(21.7) | 0(0.0) |  |
| intracapsular septation | without | 50(61) | 83(92.2) | <.001* | 18(78.3) | 19(95.0) | .255*** |
|  | with | 32(39) | 7(7.8) |  | 5(21.7) | 1(5.0) |  |
| a hypointense rim on T2WI | without | 75(91.5) | 89(98.9) | .052*** | 19(82.6) | 20(100.0) | .152*** |
|  | with | 7(8.5) | 1(1.1) |  | 4(17.4) | 0(0.0) |  |
| Intracystic nodule | without | 76(92.7) | 46(51.1) | <.001* | 22(95.7) | 12(60.0) | .013*** |
|  | with | 6(7.3) | 44(48.9) |  | 1(4.3) | 8(40.0) |  |
| The relationship with the cavernous sinus | within | 51(62.2) | 82(91.1) | <.001* | 11(47.8) | 19(95.0) | .001* |
|  | beyond | 31(37.8) | 8(8.9) |  | 12(52.2) | 1(5.0) |  |

Note: Data in parentheses are percentages.

* From the x2 test; ** From the Fisher's Exact Test; *** From the x2 test with continuity correction.
